# Supplementary material for: Lipid profiles and risk of major adverse cardiovascular events in CKD and diabetes: A nationwide population-based study
Source: PLoS One. 2020 Apr 9;15(4):e0231328. doi: 10.1371/journal.pone.0231328 (PMC7144995; doi:10.1371/journal.pone.0231328)
Supplement: S1 Table — (DOCX) [file pone.0231328.s001.docx]

S1 Table. Association of serum LDL-c with MACEs and all-cause mortality in patients with CKD and diabetes with LDL-c <79 mg/dL (1st octiles)

| **MACE** | | | **Baseline model** | | **Time-varying model** | |
| --- | --- | --- | --- | --- | --- | --- |
| Level | N | Event | HR (95% CI) | *P* value | HR (95% CI) | *P* value |
| <30 | 306 | 35 | 0.955 (0.678,1.344) | 0.79 | 0.975 (0.693,1.373) | 0.63 |
| 30-49 | 915 | 126 | 1.172 (0.967,1.42) | 0.17 | 1.133 (0.935,1.373) | 0.65 |
| 50-78 | 5343 | 660 | 1 (Ref.) | 0.25 | 1 (Ref.) | 0.81 |
| **All-cause mortality** | | |  |  |  |  |
| <30 | 306 | 63 | 1.205 (0.93,1.562) | 0.16 | 1.259 (0.972,1.631) | 0.04 |
| 30-49 | 915 | 191 | 1.319 (1.126,1.545) | <0.001 | 1.256 (1.072,1.472) | <0.001 |
| 50-78 | 5343 | 936 | 1 (Ref.) | 0.002 | 1 (Ref.) | 0.18 |
