# Supplementary material for: Lipid profiles and risk of major adverse cardiovascular events in CKD and diabetes: A nationwide population-based study
Source: PLoS One. 2020 Apr 9;15(4):e0231328. doi: 10.1371/journal.pone.0231328 (PMC7144995; doi:10.1371/journal.pone.0231328)
Supplement: S2 Table — (DOCX) [file pone.0231328.s002.docx]

S2 Table. Association of serum LDL-c with MACEs and all-cause mortality in advanced CKD patients with LDL-c <79 mg/dL (1st octiles)

| **MACE** | | | **Baseline model** | | **Time-varying model** | |
| --- | --- | --- | --- | --- | --- | --- |
| Level | N | Event | HR (95% CI) | *P* value | HR (95% CI) | *P* value |
| <30 | 66 | 9 | 1.065 (0.541,2.096) | 0.86 | 1.23 (0.625,2.42) | 0.55 |
| 30-49 | 180 | 29 | 1.299 (0.87,1.941) | 0.20 | 1.296 (0.868,1.936) | 0.21 |
| 50-78 | 1185 | 159 | 1 (Ref.) | 0.44 | 1 (Ref.) | 0.40 |
| **All-cause mortality** | | |  |  |  |  |
| <30 | 66 | 14 | 1.096 (0.637,1.887) | 0.74 | 1.255 (0.729,2.16) | 0.41 |
| 30-49 | 180 | 56 | 1.831 (1.364,2.458) | <0.001 | 1.803 (1.342,2.422) | <0.001 |
| 50-78 | 1185 | 255 | 1 (Ref.) | <0.001 | 1 (Ref.) | <0.001 |
