# Supplementary material for: Lipid profiles and risk of major adverse cardiovascular events in CKD and diabetes: A nationwide population-based study
Source: PLoS One. 2020 Apr 9;15(4):e0231328. doi: 10.1371/journal.pone.0231328 (PMC7144995; doi:10.1371/journal.pone.0231328)
Supplement: S3 Table — (DOCX) [file pone.0231328.s003.docx]

S3 Table. Association of serum LDL-c with MACE and all-cause mortality, stratified by octiles categories in CKD patients with diabetes in a statin-dropout model (for statin therapy).

| **MACE** | | | **Statin-dropout model*** | | | |
| --- | --- | --- | --- | --- | --- | --- |
|  | | | **Unadjusted** | | **Adjusted** | |
| level | N | Event | HR (95% CI) | *P* value | HR (95% CI) | *P* value |
| <79 | 6564 | 821 | 1.012 (0.901,1.136) | 0.8446 | 0.906 (0.805,1.02) | 0.1021 |
| 79-94 | 6492 | 779 | 0.972 (0.865,1.092) | 0.6283 | 0.9 (0.8,1.013) | 0.0813 |
| 95-105 | 6096 | 745 | 0.948 (0.841,1.068) | 0.3795 | 0.898 (0.796,1.014) | 0.0818 |
| 106-116 | 6693 | 847 | 1.052 (0.938,1.18) | 0.3868 | 1.044 (0.93,1.172) | 0.4676 |
| 117-127 | 6540 | 825 | 1 (Ref.) |  | 1 (Ref.) |  |
| 128-140 | 6581 | 826 | 0.976 (0.865,1.1) | 0.686 | 1.008 (0.893,1.138) | 0.8976 |
| 141-158 | 6439 | 812 | 0.988 (0.874,1.117) | 0.8492 | 1.079 (0.953,1.221) | 0.2306 |
| ≥159 | 6352 | 900 | 1.25 (1.108,1.411) | 0.0003 | 1.389 (1.228,1.571) | <.0001 |
| **All-cause mortality** | | |  |  |  |  |
| <79 | 6564 | 1190 | 1.545 (1.39,1.717) | <.0001 | 1.312 (1.177,1.462) | <.0001 |
| 79-94 | 6492 | 1058 | 1.252 (1.122,1.397) | <.0001 | 1.118 (1,1.25) | 0.0508 |
| 95-105 | 6096 | 907 | 1.186 (1.059,1.328) | 0.0031 | 1.091 (0.973,1.224) | 0.1371 |
| 106-116 | 6693 | 875 | 1.014 (0.903,1.138) | 0.8187 | 1 (0.889,1.125) | 0.9941 |
| 117-127 | 6540 | 877 | 1 (Ref.) |  | 1 (Ref.) |  |
| 128-140 | 6581 | 796 | 0.936 (0.829,1.057) | 0.2877 | 1.006 (0.889,1.138) | 0.9247 |
| 141-158 | 6439 | 784 | 0.998 (0.882,1.13) | 0.9769 | 1.159 (1.022,1.314) | 0.0218 |
| ≥159 | 6352 | 802 | 0.982 (0.861,1.12) | 0.7899 | 1.186 (1.037,1.357) | 0.0128 |

*In the statin-dropout models, an additional adjustment was also done for statin therapy (i.e., if statin therapy was ever used at any time during the follow-up period) in addition to the fully adjusted model.

Multivariate adjustment, which adjusted for baseline characteristics of age, sex, BMI, smoking, systolic blood pressure, diastolic blood pressure, eGFR, urinary protein, serum hemoglobin and glucose level.
