# Supplementary material for: Lipid profiles and risk of major adverse cardiovascular events in CKD and diabetes: A nationwide population-based study
Source: PLoS One. 2020 Apr 9;15(4):e0231328. doi: 10.1371/journal.pone.0231328 (PMC7144995; doi:10.1371/journal.pone.0231328)
Supplement: S4 Table — (DOCX) [file pone.0231328.s004.docx]

S4 Table. Association of serum LDL-c with MACE and all-cause mortality, stratified by octiles categories in early and advanced CKD with diabetes in a statin-dropout model (multivariate adjustment only).

| **MACE** | | | **Statin-dropout model** | | | |
| --- | --- | --- | --- | --- | --- | --- |
|  | | | **Early CKD** | | **Advanced CKD** | |
| level | N | Event | HR (95% CI)* | *P* value | HR (95% CI) | *P* value |
| <79 | 6564 | 821 | 0.9 (0.788,1.029) | 0.1248 | 0.933 (0.726,1.198) | 0.5846 |
| 79-94 | 6492 | 779 | 0.893 (0.78,1.022) | 0.0994 | 0.932 (0.728,1.192) | 0.5737 |
| 95-105 | 6096 | 745 | 0.903 (0.788,1.035) | 0.1434 | 0.9 (0.693,1.17) | 0.4324 |
| 106-116 | 6693 | 847 | 1.017 (0.891,1.16) | 0.8062 | 1.128 (0.886,1.437) | 0.328 |
| 117-127 | 6540 | 825 | 1 (Ref.) |  | 1 (Ref.) |  |
| 128-140 | 6581 | 826 | 0.926 (0.805,1.065) | 0.279 | 1.31 (1.024,1.677) | 0.0318 |
| 141-158 | 6439 | 812 | 1.04 (0.903,1.198) | 0.5883 | 1.184 (0.914,1.532) | 0.2008 |
| ≥159 | 6352 | 900 | 1.316 (1.142,1.516) | 0.0001 | 1.61 (1.252,2.07) | 0.0002 |
| **All-cause mortality** | | |  |  |  |  |
| <79 | 6564 | 1190 | 1.362 (1.2,1.547) | <.0001 | 1.162 (0.945,1.429) | 0.1544 |
| 79-94 | 6492 | 1058 | 1.197 (1.05,1.365) | 0.007 | 0.9 (0.725,1.118) | 0.3424 |
| 95-105 | 6096 | 907 | 1.183 (1.036,1.351) | 0.013 | 0.841 (0.667,1.061) | 0.1448 |
| 106-116 | 6693 | 875 | 1.038 (0.904,1.192) | 0.5975 | 0.873 (0.698,1.091) | 0.2324 |
| 117-127 | 6540 | 877 | 1 (Ref.) |  | 1 (Ref.) |  |
| 128-140 | 6581 | 796 | 1.023 (0.885,1.182) | 0.7619 | 0.945 (0.744,1.2) | 0.6406 |
| 141-158 | 6439 | 784 | 1.17 (1.009,1.357) | 0.0383 | 1.102 (0.869,1.397) | 0.4249 |
| ≥159 | 6352 | 802 | 1.197 (1.021,1.404) | 0.0268 | 1.102 (0.856,1.418) | 0.4502 |
