# Supplementary material for: Lipid profiles and risk of major adverse cardiovascular events in CKD and diabetes: A nationwide population-based study
Source: PLoS One. 2020 Apr 9;15(4):e0231328. doi: 10.1371/journal.pone.0231328 (PMC7144995; doi:10.1371/journal.pone.0231328)
Supplement: S5 Table — (DOCX) [file pone.0231328.s005.docx]

S5 Table. Association of serum TG/HDL-c ratio with MACE and all-cause mortality, stratified by octiles categories in CKD patients with diabetes in a statin-dropout model.

| **MACE** | | | **Statin-dropout model** | | | |
| --- | --- | --- | --- | --- | --- | --- |
|  | | | **Unadjusted** | | **Adjusted** | |
| level | N | Event | HR (95% CI) | *P* value | HR (95% CI) | *P* value |
| <1.44 | 6472 | 626 | 0.705 (0.624,0.797) | <.0001 | 0.762 (0.673,0.863) | <.0001 |
| 1.44-1.95 | 6466 | 701 | 0.8 (0.711,0.902) | 0.0003 | 0.807 (0.716,0.91) | 0.0005 |
| 1.95-2.45 | 6469 | 774 | 0.894 (0.795,1.005) | 0.0596 | 0.894 (0.794,1.006) | 0.0626 |
| 2.45-3.00 | 6488 | 825 | 0.914 (0.813,1.027) | 0.1306 | 0.908 (0.807,1.021) | 0.1064 |
| 3.00-3.72 | 6455 | 874 | 1 (Ref.) |  | 1 (Ref.) |  |
| 3.72-4.73 | 6470 | 885 | 1.003 (0.894,1.125) | 0.9568 | 1.013 (0.903,1.138) | 0.8221 |
| 4.73-6.57 | 6470 | 941 | 1.043 (0.93,1.17) | 0.4679 | 1.061 (0.945,1.192) | 0.316 |
| ≥6.57 | 6467 | 929 | 1.003 (0.892,1.127) | 0.961 | 1.075 (0.954,1.21) | 0.236 |
| **All-cause mortality** | | |  |  |  |  |
| <1.44 | 6472 | 899 | 1.021 (0.914,1.141) | 0.7122 | 1.025 (0.915,1.148) | 0.6671 |
| 1.44-1.95 | 6466 | 912 | 1.041 (0.931,1.163) | 0.4819 | 0.99 (0.883,1.109) | 0.8576 |
| 1.95-2.45 | 6469 | 973 | 1.078 (0.964,1.205) | 0.1857 | 1.037 (0.926,1.162) | 0.5241 |
| 2.45-3.00 | 6488 | 983 | 1.119 (1.001,1.25) | 0.0479 | 1.111 (0.993,1.244) | 0.0668 |
| 3.00-3.72 | 6455 | 903 | 1 (Ref.) |  | 1 (Ref.) |  |
| 3.72-4.73 | 6470 | 909 | 0.992 (0.884,1.114) | 0.8979 | 1.036 (0.921,1.164) | 0.5591 |
| 4.73-6.57 | 6470 | 877 | 0.922 (0.819,1.039) | 0.1841 | 1.01 (0.894,1.14) | 0.8757 |
| ≥6.57 | 6467 | 833 | 0.89 (0.788,1.005) | 0.0596 | 1.111 (0.98,1.258) | 0.0992 |
