# Supplementary material for: Lipid profiles and risk of major adverse cardiovascular events in CKD and diabetes: A nationwide population-based study
Source: PLoS One. 2020 Apr 9;15(4):e0231328. doi: 10.1371/journal.pone.0231328 (PMC7144995; doi:10.1371/journal.pone.0231328)
Supplement: S6 Table — (DOCX) [file pone.0231328.s006.docx]

S6 Table. Association of serum TG/HDL-c ratio with MACE and all-cause mortality, stratified by octiles categories in early and advanced CKD with diabetes in a statin-dropout model.

| **MACE** | | | **Statin-dropout model** | | | |
| --- | --- | --- | --- | --- | --- | --- |
|  | | | **Early CKD** | | **Advanced CKD** | |
| level | N | Event | HR (95% CI) | *P* value | HR (95% CI) | *P* value |
| <1.44 | 6472 | 626 | 0.777 (0.674,0.894) | 0.0005 | 0.766 (0.589,0.996) | 0.0465 |
| 1.44-1.95 | 6466 | 701 | 0.824 (0.718,0.946) | 0.0059 | 0.808 (0.628,1.039) | 0.0962 |
| 1.95-2.45 | 6469 | 774 | 0.896 (0.782,1.026) | 0.1126 | 0.906 (0.712,1.154) | 0.424 |
| 2.45-3.00 | 6488 | 825 | 0.92 (0.804,1.053) | 0.2277 | 0.875 (0.688,1.112) | 0.2747 |
| 3.00-3.72 | 6455 | 874 | 1 (Ref.) |  | 1 (Ref.) |  |
| 3.72-4.73 | 6470 | 885 | 1.066 (0.935,1.216) | 0.3395 | 0.842 (0.659,1.075) | 0.1684 |
| 4.73-6.57 | 6470 | 941 | 1.02 (0.89,1.169) | 0.7742 | 1.135 (0.905,1.423) | 0.2742 |
| ≥6.57 | 6467 | 929 | 1.066 (0.928,1.224) | 0.3661 | 1.046 (0.827,1.325) | 0.7057 |
| **All-cause mortality** | | |  |  |  |  |
| <1.44 | 6472 | 899 | 1.026 (0.9,1.169) | 0.7011 | 1.041 (0.826,1.312) | 0.735 |
| 1.44-1.95 | 6466 | 912 | 0.983 (0.862,1.121) | 0.7959 | 1.048 (0.838,1.312) | 0.6804 |
| 1.95-2.45 | 6469 | 973 | 0.997 (0.874,1.139) | 0.9693 | 1.167 (0.937,1.453) | 0.167 |
| 2.45-3.00 | 6488 | 983 | 1.102 (0.966,1.257) | 0.1474 | 1.137 (0.912,1.417) | 0.2541 |
| 3.00-3.72 | 6455 | 903 | 1 (Ref.) |  | 1 (Ref.) |  |
| 3.72-4.73 | 6470 | 909 | 1.05 (0.917,1.204) | 0.4787 | 0.997 (0.792,1.255) | 0.977 |
| 4.73-6.57 | 6470 | 877 | 1.066 (0.926,1.228) | 0.375 | 0.859 (0.677,1.091) | 0.2135 |
| ≥6.57 | 6467 | 833 | 1.071 (0.923,1.242) | 0.3665 | 1.219 (0.965,1.54) | 0.0968 |
