# Supplementary material for: Lipid profiles and risk of major adverse cardiovascular events in CKD and diabetes: A nationwide population-based study
Source: PLoS One. 2020 Apr 9;15(4):e0231328. doi: 10.1371/journal.pone.0231328 (PMC7144995; doi:10.1371/journal.pone.0231328)
Supplement: Attachment — Submitted filename: Response to Reviewers.docx [file pone.0231328.s007.docx]

Dear Editor-in-Chief,

Thank you for giving us the opportunity to revise our manuscript entitled “**Lipid profiles and risk of major adverse cardiovascular events in patients with chronic kidney disease and diabetes: A nationwide population-based study**” by Y Lee *et al*. submitted for consideration for publication in ***PLOS ONE***.

The editor’s and reviewer’s comments were very helpful and we have revised our manuscript accordingly. We have addressed critiques as follows. We believe that the revised manuscript has been significantly improved as a result of the editor’s and reviewers’ comments, and we hope that it will now be acceptable for publication in ***PLOS ONE***.

Sincerely yours,

December 26, 2019

Dong Ki Kim

Dong Ki Kim, M.D., Ph.D.

Department of Internal Medicine, Seoul National University College of Medicine, 103 Daehakro, Jongno-gu, Seoul, 03080, Korea

Tel: 82-2-2072-2303, Fax: 82-2-745-2264

E-mail: dkkim73@gmail.com

Reviewer comments:

Reviewer #1:

1) title, please don't use "patients"

🡪 We agree with the review’s comments. As the reviewer recommended, we have removed “patients” from the title. The corrected title is as below:

*Lipid profiles and risk of major adverse cardiovascular events in CKD and diabetes: A nationwide population-based study*

2) abstract, please provide numeric values (eg, OR values and 95% CIs)

🡪 We thank the reviewer for critical comments. We have added P-for-trend for the linear association of LDL-c or TG/HDL-c ratio with HR, and we also added the lowest and highest HR (95% CI) for the U-shape relationship in the revised manuscript. The sentences added in the abstract are the following.

*There was a positive linear association between serum LDL-c and MACE risk in both early and advanced CKD patients (P <0.001 for trend). A U-shaped relationship was observed between serum LDL-c and all-cause mortality (the 4th and 8th octile groups; lowest hazard ratio [HR] 0.96, 95% confidence interval [CI] 0.87-1.05 and highest HR 1.14, 95% CI 1.04-1.26, respectively). A similar pattern remained in both early and advanced CKD patients. The TG/HDL-c ratio categories showed a positive linear association for MACE risk in early CKD (P <0.001 for trend),*

3) Table 1, please remove P-value due to large sample size

🡪 Thank you for the reviewer’s thoughtful comments. We have removed P-values in Table 1.

4) The lowest cutoff point for LDL was 79 mg/dL. Please further look at 50 and 30 mg/dL due to the large case number

🡪 We thank the reviewer for these points. As the reviewer recommended, we have looked further at patients with LDL-c <79 mg/dL (1^st^ octiles). Of 6,564 patients, the number of patients with the categories of LDL-c 50-78 mg/dL and 30-49 mg/dL was 5,343 and 915, respectively. Most of the 1st octile group of baseline LDL-c had LDL-c levels above 50 mg/dL, and those with extremely low LDL-c (<30 mg/dL) accounted for 0.6% of the total (306/51,757), which were very low, resulting in insufficient statistical power.

5) Please discuss the potential limitation for using ICD codes to identify cases.

🡪 We agree with the reviewer’s comment on this. The International classification of diseases (ICD) data are often used by health researchers to study health services, mortality, and other outcomes. Although validation studies can be carried out to ensure the validity of coding in administrative data, there are still limitations when using ICD information (Izeta Kurbasic et al. Acta Inform Med. 2008; 16(3): 159–161., Jetté et al. Med Care 2010;48: 1105-1110). The ICD classification is not suitable if there is few or no information about the patient. In this case, we can only code symptoms of the disease, which can be caused by several different health conditions that can be coded regularly, if we have enough information to confirm diagnosis. Consequently, ICD-based diagnosis may underestimate the true prevalence of diseases. Also, disease severity can usually not be determined using ICD-coded data alone. In a recent report, it was concluded that the ICD codes alone failed to capture many heart failure (HF) admissions, and that using the hospital codes alone would underestimate the total burden of HF (E. Ingelsson et al. The European Journal of Heart Failure (2005) 787–791). This limitation was also reported in the identification of diabetes (Khokhar B, et al. BMJ Open 2016;6:e009952). We have added this issue as a limitation in the revised manuscript. Then added sentence and rearranged the sentences in the revised discussion is the following:

*Second, prevalence of diseases could be underestimated since we used ICD-based diagnosis(34-36). As the presence of diabetes was defined based on the prescription of antidiabetic medication under ICD-10 codes E11–14 or fasting glucose levels ≥ 126 mg, subjects with undiagnosed diabetes or those who did not visit a hospital during the study period could be omitted from these data. Also, disease severity can usually not be determined using ICD-coded data alone.*

Reviewer #2: Using the Korean National Health Insurance Database in this manuscript the authors have described the association of serum lipid parameters with cardiovascular outcomes in a large population of Korean patients with mild to severe diabetic nephropathy.

They found that patients with advanced CKD (eGFR <30 mL/min/1.73 m2) had lower serum total cholesterol, LDL-c, and HDL-c but higher non-HDL-c levels and triglyceride/HDL-c ratios. They found a positive correlation between serum LDL-c and the major adverse cardiovascular events (MACE) in patients with early and advanced CKD. They further found a U-shaped relationship between serum LDL-c and all-cause mortality. The TG/HDL-c ratio showed a positive association for risk of MACE in patients with early CKD, but not advanced CKD. They found no correlation between serum TG/HDL ratio and all-cause mortality in the study population. The LDL-c level predicted the risk for MACEs and all-cause mortality in diabetic patients with early and advanced CKD, although the patterns of the association differed from each other. The TG/HDL-chol ratio did not predict the risk for either MACEs or all-cause mortality in patients with advanced diabetic nephropathy. However, the TG/HDL-c ratio predicted the risk of MACE in diabetic patients with early CKD.

The data presented on a large Korean CKD population in this paper is novel and consistent with those reported on CKD populations of other nationalities.

🡪 We appreciate the reviewer for the thoughtful comment. Our study aimed to identify possible associations between lipid profile parameters and MACEs and mortality in CKD patients with diabetes and to evaluate the impact of kidney function on the associations, with large cohort and inclusiveness. Accordingly, we believe that the present data showed the consistence with those reported on CKD with diabetes populations of other nationalities.

Again, we would like to thank the reviewer very much.
