# Supplementary material for: Lipid profiles and risk of major adverse cardiovascular events in CKD and diabetes: A nationwide population-based study
Source: PLoS One. 2020 Apr 9;15(4):e0231328. doi: 10.1371/journal.pone.0231328 (PMC7144995; doi:10.1371/journal.pone.0231328)
Supplement: Attachment — Submitted filename: Response to Reviewers_revised2 (1).docx [file pone.0231328.s008.docx]

Dear Editor-in-Chief,

Thank you for giving us the opportunity to revise our manuscript entitled “**Lipid profiles and risk of major adverse cardiovascular events in chronic kidney disease and diabetes: A nationwide population-based study**” by Y Lee *et al*. submitted for consideration for publication in ***PLOS ONE***.

The editor’s and reviewer’s comments were very helpful, and we have revised our manuscript accordingly. We have addressed critiques as follows. We believe that the revised manuscript has been significantly improved as a result of the editor’s and reviewers’ comments, and we hope that it will now be acceptable for publication in ***PLOS ONE***.

Sincerely yours,

February 18, 2020

Dong Ki Kim

Dong Ki Kim, M.D., Ph.D.

Department of Internal Medicine, Seoul National University College of Medicine, 103 Daehakro, Jongno-gu, Seoul, 03080, Korea

Tel: 82-2-2072-2303, Fax: 82-2-745-2264

E-mail: dkkim73@gmail.com

Reviewer comments:

Reviewer #1: Thanks for addressing most of my concerns. However, there were still ~1200 participants with LDL <50 and 300 with LDL <30. You may have power to detect a trend or moderate-to-big difference. Statistical significance is not a major issue we should concern about. Even you did not find statistically significant results, it is still worthy to report these results as they could be practically significant.

🡪 We thank the reviewer for these points, again. As the reviewer recommended, we have looked further at patients with LDL-c <79 mg/dL (1^st^ octiles) and additionally analyzed subgroups with the categories of LDL-c 50-78 mg/dL, 30-49 mg/dL and <30 mg/dL. Most of the 1st octile group of baseline LDL-c had LDL-c levels above 50 mg/dL, and those with extremely low LDL-c (<30 mg/dL) accounted for 0.6% of the total (306/51,757). When compared with patients with LDL-c 50-78 mg/dL, the relative hazard of MACEs and all-cause mortality in the category of LDL-c 30-49 mg/dL was higher in both baseline and time-varying models (highest HR 1.32; 95% CI 1.13-1.55). Notably, this trend was more prominent in patients with advanced CKD (HR 1.83; 95% CI 1.36-2.45 and HR 1.80; 95% CI 1.34-2.42 in baseline and time-varying models, respectively). These additional results more firmly suggested that the shape of association between serum LDL-c and all-cause mortality is a U-curve, especially in patients with advanced CKD, which is considered to support real-world data. We have added these results in the result section of the revised manuscript. The summarized tables of the association of serum LDL-c <79 mg/dL with MACEs and all-cause mortality are below.

Association of serum LDL-c with MACEs and all-cause mortality in patients with CKD and diabetes with LDL-c <79 mg/dL (1^st^ octiles)

| **MACE** | | | **Baseline model** | | **Time-varying model** | |
| --- | --- | --- | --- | --- | --- | --- |
| Level | N | Event | HR (95% CI) | *P* value | HR (95% CI) | *P* value |
| <30 | 306 | 35 | 0.955 (0.678,1.344) | 0.79 | 0.975 (0.693,1.373) | 0.63 |
| 30-49 | 915 | 126 | 1.172 (0.967,1.42) | 0.17 | 1.133 (0.935,1.373) | 0.65 |
| 50-78 | 5343 | 660 | 1 (Ref.) | 0.25 | 1 (Ref.) | 0.81 |
| **All-cause mortality** | | |  |  |  |  |
| <30 | 306 | 63 | 1.205 (0.93,1.562) | 0.16 | 1.259 (0.972,1.631) | 0.04 |
| 30-49 | 915 | 191 | 1.319 (1.126,1.545) | <0.001 | 1.256 (1.072,1.472) | <0.001 |
| 50-78 | 5343 | 936 | 1 (Ref.) | 0.002 | 1 (Ref.) | 0.18 |

Association of serum LDL-c with MACEs and all-cause mortality in advanced CKD patients with LDL-c <79 mg/dL (1^st^ octiles)

| **MACE** | | | **Baseline model** | | **Time-varying model** | |
| --- | --- | --- | --- | --- | --- | --- |
| Level | N | Event | HR (95% CI) | *P* value | HR (95% CI) | *P* value |
| <30 | 66 | 9 | 1.065 (0.541,2.096) | 0.86 | 1.23 (0.625,2.42) | 0.55 |
| 30-49 | 180 | 29 | 1.299 (0.87,1.941) | 0.20 | 1.296 (0.868,1.936) | 0.21 |
| 50-78 | 1185 | 159 | 1 (Ref.) | 0.44 | 1 (Ref.) | 0.40 |
| **All-cause mortality** | | |  |  |  |  |
| <30 | 66 | 14 | 1.096 (0.637,1.887) | 0.74 | 1.255 (0.729,2.16) | 0.41 |
| 30-49 | 180 | 56 | 1.831 (1.364,2.458) | <0.001 | 1.803 (1.342,2.422) | <0.001 |
| 50-78 | 1185 | 255 | 1 (Ref.) | <0.001 | 1 (Ref.) | <0.001 |

The sentences added in the revised result are the following.

*Next, we conducted subgroup analyses to detect the effect of LDL-c levels on MACE risk and mortality in patients with extremely low LDL-c (<79 mg/dL), by dividing into three subgroups of LDL-c 50-78 mg/dL, 30-49 mg/dL and <30 mg/dL. When compared with patients with LDL-c 50-78 mg/dL, the relative hazard of MACEs and all-cause mortality in the category of LDL-c 30-49 mg/dL was higher in both baseline and time-varying models (highest HR 1.32, 95% CI 1.13-1.55). Notably, this trend was more prominent in patients with advanced CKD (HR 1.83, 95% CI 1.36-2.45 and HR 1.80, 95% CI 1.34-2.42 in baseline and time-varying models, respectively).*

We would like to thank the reviewer for this helpful comment.

Reviewer #2: The authors have addressed the issues I had raised satisfactorily. I therefore recommend acceptance of their paper for publication.

🡪 We are grateful for the reviewer’s thoughtful comments. Our study aimed to identify possible associations between lipid profile parameters and MACEs and mortality in CKD patients with diabetes and to evaluate the impact of kidney function on the associations, with large cohort and inclusiveness. Accordingly, we believe that the present data showed the consistence with those reported on CKD with diabetes populations of other nationalities.

Again, we would like to thank the reviewer very much.
