# Supplementary material for: Lipid profiles and risk of major adverse cardiovascular events in CKD and diabetes: A nationwide population-based study
Source: PLoS One. 2020 Apr 9;15(4):e0231328. doi: 10.1371/journal.pone.0231328 (PMC7144995; doi:10.1371/journal.pone.0231328)
Supplement: Attachment — Submitted filename: Response to Reviewers_revised3.docx [file pone.0231328.s009.docx]

Dear Editor-in-Chief,

Thank you for giving us the opportunity to revise our manuscript entitled “**Lipid profiles and risk of major adverse cardiovascular events in chronic kidney disease and diabetes: A nationwide population-based study**” by Y Lee *et al*. submitted for consideration for publication in ***PLOS ONE***.

The editor’s and reviewer’s comments were very helpful, and we have revised our manuscript accordingly. We have addressed critiques as follows. We believe that the revised manuscript has been significantly improved as a result of the editor’s and reviewers’ comments, and we hope that it will now be acceptable for publication in ***PLOS ONE***.

Sincerely yours,

February 27, 2020

Dong Ki Kim

Dong Ki Kim, M.D., Ph.D.

Department of Internal Medicine, Seoul National University College of Medicine, 103 Daehakro, Jongno-gu, Seoul, 03080, Korea

Tel: 82-2-2072-2303, Fax: 82-2-745-2264

E-mail: dkkim73@gmail.com

Reviewer comments:

Reviewer #1: Thank you for including additional analysis to look at those with low LDL-c concentration. Results are important for public health and clinical practice. The only suggestion is to add these results into your abstract. This is important because in abstract you stated that "There was a positive linear association between serum LDL-c and MACE risk in both early and advanced CKD patients (P <0.001 for trend)" (Line 42). This may not be true based on your additional analysis: "the relative hazard of MACEs and all-cause mortality in the category of LDL-c 30-49 mg/dL was higher in both baseline and time-varying models" (line 250-251).

🡪 Thank you for the reviewer’s thoughtful comments. We have added these results in the abstract. The sentences added in the abstract are the following.

*There was a positive linear association between serum LDL-c and MACE risk in both early and advanced CKD patients (P <0.001 for trend), except for the category of LDL-c 30-49 mg/dL in extremely low LDL-c subgroup analyses.*

Again, we would like to thank the reviewer very much.
